# Disruption of *rcsB* by a duplicated sequence in a curli-producing *Escherichia coli* O157:H7 results in differential gene expression in relation to biofilm formation, stress responses and metabolism

**DOI:** 10.1186/s12866-017-0966-x

**Published:** 2017-03-08

**Authors:** V. K. Sharma, D. O. Bayles, D. P. Alt, T. Looft, B. W. Brunelle, J. A. Stasko

**Affiliations:** 1 Food Safety and Enteric Pathogens Research Unit, National Animal Disease Center, ARS-USDA, P. O. Box 70, 1920 Dayton Avenue, Ames, IA 50010 USA; 2 Infectious Bacterial Diseases Research Unit, National Animal Disease Center, ARS-USDA, Ames, IA 50010 USA; 3 Microscopy Services Unit, National Animal Disease Center, ARS-USDA, Ames, IA 50010 USA

**Keywords:** EHEC, Stress signaling, Curli, Biofilms

## Abstract

**Background:**

*Escherichia coli* O157:H7 (O157) strain 86–24, linked to a 1986 disease outbreak, displays curli- and biofilm-negative phenotypes that are correlated with the lack of Congo red (CR) binding and formation of white colonies (CR^−^) on a CR-containing medium. However, on a CR medium this strain produces red isolates (CR^+^) capable of producing curli fimbriae and biofilms.

**Results:**

To identify genes controlling differential expression of curli fimbriae and biofilm formation, the RNA-Seq profile of a CR^+^ isolate was compared to the CR^−^ parental isolate. Of the 242 genes expressed differentially in the CR^+^ isolate, 201 genes encoded proteins of known functions while the remaining 41 encoded hypothetical proteins. Among the genes with known functions, 149 were down- and 52 were up-regulated. Some of the upregulated genes were linked to biofilm formation through biosynthesis of curli fimbriae and flagella. The genes encoding transcriptional regulators, such as CsgD, QseB, YkgK, YdeH, Bdm, CspD, BssR and FlhDC, which modulate biofilm formation, were significantly altered in their expression. Several genes of the envelope stress (*cpxP*), heat shock (*rpoH*, *htpX*, *degP*), oxidative stress (*ahpC*, *katE*), nutrient limitation stress (*phoB-phoR* and *pst*) response pathways, and amino acid metabolism were downregulated in the CR^+^ isolate. Many genes mediating acid resistance and colanic acid biosynthesis, which influence biofilm formation directly or indirectly, were also down-regulated. Comparative genomics of CR^+^ and CR^−^ isolates revealed the presence of a short duplicated sequence in the *rcsB* gene of the CR^+^ isolate. The alignment of the amino acid sequences of RcsB of the two isolates showed truncation of RcsB in the CR^+^ isolate at the insertion site of the duplicated sequence. Complementation of CR^+^ isolate with *rcsB* of the CR^−^ parent restored parental phenotypes to the CR^+^ isolate.

**Conclusions:**

The results of this study indicate that RcsB is a global regulator affecting bacterial survival in growth-restrictive environments through upregulation of genes promoting biofilm formation while downregulating certain metabolic functions. Understanding whether *rcsB* inactivation enhances persistence and survival of O157 in carrier animals and the environment would be important in developing strategies for controlling this bacterial pathogen in these niches.

## Background

*Escherichia coli* O157:H7 (O157) is a frequent cause of foodborne disease outbreaks, resulting primarily through the consumption of contaminated bovine food products, water and fresh produce [1]. Similar to many pathogenic and non-pathogenic *E. coli* strains, O157 encounters a variety of growth conditions when present as a transient or long-term colonizer of the host animal gastrointestinal tract or living in the environment external to the animal host [2]. The ability of O157 to adapt to and survive in diverse conditions is contingent upon rapidly sensing and responding to specific cues in order to express genetic programs suited for energy conservation, growth and survival in a specific environmental niche [3–6]. The formation of biofilms represents a survival strategy involving intricate network of regulatory circuits controlling induction of various pathways conducive for biofilm formation [7, 8]. Some of these pathways encode structural elements such as curli fimbriae, cellulose and colanic acid that play specific roles at various stages of biofilm formation [9, 10]. Curli fimbriae, which are highly adhesive equivalents of functional amyloids and encoded by the divergently transcribed *csgBAC* and *csgDEFG* operons, are important in biofilm formation by promoting initial bacterial-substratum interactions and subsequent cell-cell aggregation [7].

The *csgA* gene of the *csgBAC* operon encodes for curlin, which is a major structural protein of curli fimbriae [10]. Curli fimbriae have a high affinity for Congo red and enable curli-positive O157 bacterial cells to produce red colonies compared to the white colonies produced by curli-negative bacterial cells on a Congo red containing agar medium [10, 11]. One of the key elements of the regulatory networks controlling curli expression is CsgD, which is a member of the FixJ/LuxR/UhpA family of transcriptional regulators. CsgD governs transition of *E. coli* from planktonic to biofilm mode of existence [9].

Since CsgD is a critical transcriptional regulator of the genes encoding curli fimbriae, the expression of *csgD* is under the control of various stress signaling systems. *E. coli* encodes more than 30 two-component signal transduction (TCST) pathways to sense and respond to changes in the immediate growth environment [12]. Multiple TCST pathways are normally activated in response to a single or multiple stress signals resulting in a complex response encompassing global changes in gene expression suitable to cope with a specific stress signal. The EnvZ-OmpR TCST system activates *csgD* expression in response to low osmolarity, which serves as a cue for bacteria encountering nutrient-limiting environments [13, 14]. Increased expression of CsgD then promotes production of curli fimbriae that are essential for stable bacterial interactions with abiotic surfaces to initiate biofilm formation [7]. The response regulator RscB of the RcsCBD pathway, in conjunction with an auxiliary transcription factor RcsA, induces biosynthesis of colanic acid, which contributes to later stages of biofilm formation [15]. However, RcsB represses expression of genes encoding curli fimbriae in response to cell-envelope perturbations and changes in the divalent ion concentration [16]. The negative regulation of biofilm formation is relieved in strains carrying deletions or mutations in the *rcsB* gene with concomitant reduction in acid resistance [17]. Genes encoding bacterial flagella are also under the negative regulation of the RcsCBD phosphorelay system [18]. The *cpxRA* encoded TCST is activated in response to high pH and overproduction and misfolding of cell envelope proteins [19, 20]. These stimuli induce the autophosphorylation of CpxA, a transmembrane sensor with histidine kinase activity, which then activates the response regulator CpxR. The activated CpxR modulates the expression of CpxP, a periplasmic chaperon, facilitating misfolded protein degradation as well as repressing CpxA activity in the absence of specific stimuli mentioned above [21]. However, mutations inhibiting function of CpxA have been shown to enhance biofilm formation in *E. coli* [22]. Heat shock response is activated when *E. coli* is exposed to growth temperatures above 30 °C and generally involves activation of sigma factors RpoE and RpoH each regulating 90 and 30 genes, respectively [23–25]. Some of the proteins induced following heat shock include the DnaK-DnaJ-GrpE group of chaperones and several proteases such as DegP, HslJ and HtpX [26, 27]. The major functions of these chaperons and proteases are to prevent aggregation of proteins, promote refolding of aggregated proteins to normal conformation, or facilitate degradation of irreversibly damaged proteins resulting from exposure to high temperatures. A recent study has demonstrated that the heat shock induced chaperone DnaJ may play a role in biofilm formation as the *dnaJ* mutant produced lower amount of biofilm upon exposure to high temperatures during early stages of biofilm formation [28].

Several transcription regulators, including IHF, RpoS, MrlA, Crl, Hha, OmpA and H-NS, also control curli expression in response to various environmental signals. The stationary phase sigma factor RpoS directly activates transcription of the *csgBAC* operon in response to growth-limiting conditions and other stresses [9, 29]. The Crl protein, which is preferentially expressed at low temperatures, interacts with RpoS to activate transcription of the *csgBAC* operon [30]. Multiple transcription factors, such as integration host factor IHF, H-NS, OmpR and Hha, interact cooperatively to form a ribonucleoprotein complex with the *csgD* promoter, resulting in elevated expression under microaerophilic growth conditions [13, 31, 32]. The regulatory nucleotide c-di-GMP, which is produced in response to complex regulatory cues, activates the production of curli fimbriae and cellulose in certain *E. coli* strains [33]. Curli fimbriae and cellulose together produce a strong biofilm matrix facilitating bacterial attachment to hydrophilic and hydrophobic surfaces [8, 34].

Expression of curli fimbriae and the ability to produce biofilms are highly variable among the pathogenic *E. coli* strains, including O157 isolates of human and bovine origin [17, 35, 36]. Curli production in *E. coli* strains is positively correlated with the amount of the Congo red (CR) dye bound and biofilm biomass produced by bacterial cells. Curli-producing *E. coli* strains form red colonies (CR^+^) on CR agar medium and produce higher biofilm biomass compared to the strains that do not produce or are poor producers of curli fimbriae. These later strains form white colonies (CR^−^) on CR agar medium and do not produce biofilms. O157 strain 86–24, linked to a foodborne disease outbreak in 1986, is a poor producer of curli fimbriae and, therefore, exhibits CR^−^ phenotype and produces very low levels of biofilms [37]. However, the CR^−^ O157 strain 86–24 can give rise to CR^+^ isolates at a low frequency when grown on a CR agar medium. These CR^+^ isolates produce abundant curli fimbriae and higher biofilm biomass compared to the parental CR^−^ 86–24 isolates. In the present study, we compared the transcriptome of a CR^+^ isolate to a CR^−^ isolate of the O157 strain 86–24 in order to identify regulatory networks and pathways responsible for the curli-positive phenotype of the CR^+^ isolate. In addition, we mined the complete genome sequences of both strains to identify gene (s) responsible for the CR^+^ phenotype. Anticipated implications of these findings would be the identification of mechanisms and signals that guide this transition from CR^−^ to CR^+^ phenotype and the relative contribution of this phenotypic transition in carriage, transmission and survival of O157 in carrier animals and in the environment.

## Methods

### Bacterial strains, culture media and growth conditions

All *Escherichia coli* O157:H7 (O157) strains used in this study were derived from a streptomycin-resistant isolate of *E. coli* O157:H7 strain 86–24, originally linked to a 1986 foodborne disease outbreak in Walla Walla (Washington). *E. coli* TOP10 and plasmid pCRXL were used as a host and a vector, respectively, for initial cloning and propagation of recombinant plasmids (Invitrogen, Carlsbad, CA). Bacterial strains were cultivated in Luria-Bertani broth (LB) with or without sodium chloride (LB-No Salt). LB-agar (1.5% final concentration) was used for bacterial plate cultures. Antibiotics were added to liquid or solid media as needed (streptomycin 100 mg per liter; kanamycin 50 mg per liter).

### Isolation of Congo red-binding variants

O157 srain 86–24 was grown overnight in a culture medium prepared by mixing yeast extract (0.1%) and casamino acids (1%) in deionized water (YESCA broth). Antibiotics were used as needed. A 10 μl volume of this culture containing approximately 10^6^ colony-forming units (CFU) was streaked on YESCA agar plates (YESCA broth containing 1.5% of Noble Agar; (Daigger, Vernon Hills, IL)) containing Congo red (40 μg/ml) and Coomassie Brilliant Blue-G250 (6.25 μg/ml) (YESCA-CR). The red colonies that grew on YESCA-CR plates after 48 h of incubation at 28 °C were counted. Representative isolates of white- (O157 strain NADC 6564) and red (O157 strain NADC 6565) colonies were selected as CR^−^ parental and CR^+^ mutant isolates, respectively, for the further studies.

### Determination of the effect of temperature and culture media on bacterial growth

Bacterial growth rates were determined as per the procedure reported previously [32]. Briefly, overnight bacterial cultures, diluted (1:100) in YESCA broth or Dulbecco’s Modified Eagle’s Medium containing 0.1% glucose (DMEM; Life Technologies, Grand Island, NY), were grown at 28 °C or 37 °C in an automated growth curve reader (Growth Curves USA, Piscataway, NJ). Optical densities collected at 600_nm_ (OD_600_) by the instrument every 60 min over a 24 h period were used for generating growth plots (GraphPad Software, Inc., La Jolla, CA) and for calculating bacterial generation or doubling times (Doubling Time = *ln 2*/*ln* ODt_1_ – *ln* ODt_0_/(t_1_-t_0_)).

### Recombinant DNA procedures

Plasmids pSM757 and pSM759 were constructed for complementation of the *rcsB* gene function in strain NADC 6565. Briefly, 3.7 kb and 1.43 kb DNA fragments encoding the *rcsDB* operon and the *rcsB* gene, respectively, were amplified by PCR using the genomic DNA of strain NADC 6564 as a template. The genomic DNA was isolated by using the DNAeasy Kit (Qiagen, Valencia, CA). PCR was performed using the FailSafe PCR Kit (Epicenter, Madison, WI) and primers specific for the amplification of the *rscDB* operon (forward primer GATCACTCTAGAATTATTTTCGTTGGGCTTTTTGTAG and reverse primer GATCACTCTAGAACGCGTCTTATCTGGCCTAC) and the *rscB* gene (forward primer GATCACTCTAGAATGTTACCTCGGCAGAAATTCG and reverse primer GATCACTCTAGAACGCGTCTTATCTGGCCTAC). The PCR amplified fragments were resolved on an agarose gel by electrophoresis and following staining of the gel with ethidium bromide, the two fragments were extracted from the gel using a Gel Extraction Kit (Qiagen, Valencia, CA). The gel-extracted fragments were ligated at the TA cloning site of the cloning vector pCRXL (Invitrogen, Grand Island, NY). The ligated DNA was electroporated into *E. coli* TOP10 electrocompetent cells using a MiniPulser electroporation system and according to the manufacturer’s instructions (BIO-RAD, Hercules, CA). The resulting recombinant plasmids pSM757 (pCRXL-*rcsDB*) and pSM759 (pCRXL-*rcsB*) were purified and electroporated into the CR^−^ mutant strain NADC 6565. The empty vector pCRXL was also electroporated into CR^−^ parental (NADC 6564) and CR^+^ isolates (NADC 6565) so that these would serve as controls in subsequent experiments.

### Congo red binding assay

A previously described procedure [32] was used for comparing Congo red binding ability of bacterial strains grown for 24–48 h at 28 °C on Congo red-supplemented YESCA agar plates.

### Visualization of curli fimbriae by transmission electron microscopy

Curli fimbriae were detected using a previously described procedure involving examination of glutaraldehyde-fixed bacterial cells from a 48 h-old culture by transmission electron microscopy [32].

### Biofilm quantification

Quantification of biofilms produced after 48 h of bacterial growth in 96-well polystyrene plates was performed by using a crystal violet staining procedure described previously [32].

### Determination of swimming motility

Bacterial strains were grown overnight in LB broth plus needed antibiotics at 37 °C on a shaker-incubator (200 rpm). Aliquots (2 μl) were spotted on a soft motility agar medium (0.1% tryptone, and 25 mM sodium chloride, 0.30% noble agar) and incubated at 37 °C for about 8 h followed by incubation at 28 °C for another 16 h. Photographs of motility zones produced around the spot of inoculation were captured by photographing using the AlphaImager System (ProteinSimple, Wallingford, CT).

### Determination of sensitivity to elevated temperature, pH, osmotic and oxidative stresses

Bacterial strains were grown overnight in LB-No Salt broth at 28 °C with shaking (200 rpm). *For temperature stress*, aliquots (5 μl) of overnight cultures were diluted 1:1000 into potassium phosphate buffer (10 mM, pH 7.0). A 100 μl aliquot was taken immediately and 10-fold serial dilutions (10^−1^–10^−5^) of this aliquot were streak-plated on LB agar containing kanamycin (50 μg per ml) for determining bacterial counts at 0 min. The remainder of the culture was incubated at 55 °C, aliquots were withdrawn at 15 min, 30 min, and 60 min intervals, and10-fold serial dilutions of these aliquots were plated as above on LB agar-kanamycin plates. The plates were incubated at 37 °C and bacterial colonies were enumerated after 24 h of incubation to determine percentage survival. *For osmotic stress*, overnight bacterial cultures grown as above were diluted 1:1000 in LB-high salt (2.5 M sodium chloride). Aliquots (100 μl) were withdrawn immediately (0 min sample) and one set of cultures was incubated at 28 °C and the other at 37 °C. Aliquots (100 μl) were taken at 15 min, 30 min, and 60 min intervals. All timed samples were 10-fold serially diluted and plated on LB agar-kanamycin plates as described above. After incubation at 37 °C for 24 h, bacterial colonies were enumerated to determine percentage survival. *For oxidative stress*, 50 μl of overnight cultures grown in LB-no salt as described above were diluted 1:100 in potassium phosphate buffer (10 mM, pH 7.0). Aliquots (100 μl) were withdrawn immediately (0 min sample) and 3% hydrogen peroxide was added to the remaining cultures to a final concentration of 12.5 mM. One set of cultures was incubated at 28 °C and the other at 37 °C. Aliquots (100 μl) were withdrawn at 15 min, 30 min, and 60 min intervals. All timed samples were diluted in10-fold serial dilutions and plated on a LB agar-kanamycin plates. Bacterial colonies were enumerated on these plates after 24 h of incubation at 37 °C as described above. *For acid resistance*, bacterial cultures grown overnight in LB-no salt (pH 5.5) at 37 °C (170 rpm) were diluted 1:1000 in LB-no salt (pH2.5). Aliquots (100 μl) were withdrawn immediately (0 min sample) and the one set of cultures was incubated at 28 °C and the other at 37 °C. Aliquots (100 μl) were withdrawn at 2 h, 4 h, and 6 h intervals. All timed samples were diluted in 10-fold serial dilutions and plated on LB agar-kanamycin as described above. Bacterial colonies were enumerated after 24 h of incubation of these plates for determining percentage survival.

### Isolation of total bacterial RNA and preparation of rRNA-free RNA

Bacterial cultures (three biological replicates per bacterial strain) grown overnight in YESCA broth at 28 °C were diluted 1:100 in DMEM and grown aerobically by shaking (190 rpm) at 37 °C to the early stationary phase. One-ml aliquots of these cultures were treated with the RNA protect reagent according to the instructions of the manufacturer (Qiagen, Valencia, CA) and stored at −80 °C following the treatment. Total RNA was isolated from thawed frozen cell pellets using the RNAeasy Mini Kit according to the manufacturer’s instructions (Qiagen). Ribosomal RNA (rRNA) was removed from the total bacterial RNA using the Ribo-Zero-rRNA removal kit according to the manufacturer’s instructions (Epicentre, Madison, WI). The rRNA-depleted RNAs of CR^−^ parental (NADC 6564) and CR^+^ mutant (NADC 6565) isolates were used in the preparation of strand-specific RNA-seq libraries. These libraries were subjected to a100-bp single read sequencing with the Illumina HiSeq 2500 at the Iowa State University (Ames, IA) DNA core facility. The reads were mapped to a reference genome (O157 EDL933) and analyzed for differential gene expression.

### Transcriptomic analysis

The initial quality of the sequencing reads was assessed using FastQC [38]. Reads were trimmed using Trimmomatic [39], and the quality of the reads after trimming was determined by again analyzing the reads with FastQC. The Bowtie aligner [40] was used to map trimmed reads to the *Escherichia coli* O157:H7 EDL933 genome (NCBI accession NC_002655). Samtools was used to convert the bowtie outputs to a format amenable to counting [41]. The counts per gene were calculated by processing the mapped sequence alignments though HTSeq-count [42]. DESeq2 was used to perform the differential expression analysis [43]. The count file data for all the samples were transformed using a regularized log transformation and then analyzed by clustering and visualization of the clustering via principal component analysis (PCA) and multi-dimensional scaling (MDS) to determine whether any of the samples were outliers due to uncontrolled experimental errors. Outlier samples were subsequently removed from the analysis. The count data for the samples passing all the quality control steps were loaded into DESeq2, the contrasts of interest were specified, the differential expression (fold log_2_ difference) was calculated, and the results were filtered to limit the false discovery rate (FDR) to 10%. Fold log_2_ values were converted to fold-change values. The genes with differential expression levels of ≥ 2.0 and *p* < 0.05 were assigned to functional categories using the RAST Server, which makes predictions about the types of subsystems represented in the annotated genomes and uses this information to construct genome-specific metabolic networks [44].

### Nucleotide and amino acid sequence alignments

The complete genome sequences have been deposited at GenBank under the assigned accession numbers CP017251 (chromosome) and CP017252 (pO157) for NADC 6564 and CP017249 (chromosome) and CP017250 (pO157) for NADC 6565. The methods used for sequencing, assembling and annotation of these two genomes have been published elsewhere [45]. A comparison of the two genomes was carried out using the Artemis Comparison Tool [46]. Alignments of the nucleotide and amino acid sequences of the specific genes and proteins encoded by these genes, respectively, were completed using the Two Sequence Alignment Programs of the DNAMAN software (Lynnon Corp., Quebec, Canada).

## Results

### Congo red-positive isolates were recovered at low frequency

Streak-plating of the overnight cultures of strain 86–24 on YESCA-Congo red plates resulted in recovery of about 3 Congo red-positive colonies (CR^+^) (strain NADC 6565) per 10^6^ white colonies (strain NADC 6564) after 48 h of incubation at 28 °C.

### Increased Congo red binding of the CR^+^ isolates correlated with the production of higher biofilm biomass and curli fimbriae

**Fig. 1:**
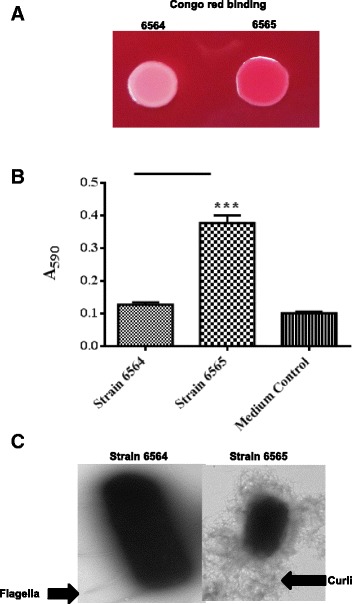
Congo red binding, biofilm production and cell surface expression of curli fimbriae by CR^−^ parental (NADC 6564) and CR^+^ mutant (NADC 6565) isolates. **a** *Color photograph* showing difference in the ability of CR^−^ and CR^+^ isolates to bind Congo red after 48 h of growth at 28 °C on YESCA agar containing Congo red. **b** Quantitative analysis of the amount of biofilms produced by strains NADC 6564 and NADC 6565 after 48 h of growth. The amount of biofilms produced was inferred from the amount of crystal violet bound by the heat-fixed biofilms. *Bars* represent the means of three independent assays and error bars represent standard deviation of 2. The * above the *bars* indicates *p* < 0.05 when NADC 6565 was compared to the parental strain NADC 6564. **c** Transmission electron micrographs of glutaraldehyde-fixed bacterial cells for detection of cell surface curli fimbriae in NADC 6564 and NADC 6565. *Dark-stained structures* represent bacterial cells and curli fimbriae appear as hair like structures (indicated by an *arrow*) on dark-stained cells. The bacterial cells were photographed at different magnifications to capture differences in the presence of curli at their cell surfaces. The strains otherwise have similar bacterial cell sizes

Fig. 1a affirms increased Congo red-binding ability of strain NADC 6565 that was originally selected as red colonies among the majority of the white colonies (NADC 6564) produced by plating strain 86–24 on a Congo red agar medium. The increased Congo red-binding ability was associated with the production of significantly higher biofilm biomass (Fig. 1b) and the formation of abundant curli fimbriae (Fig. 1c) by strain NADC 6565 compared to the CR^−^ parental strain NADC 6564.

### CR^+^ isolate showed slightly reduced growth rate compared to the CR^−^ parental strain

**Fig. 2:**
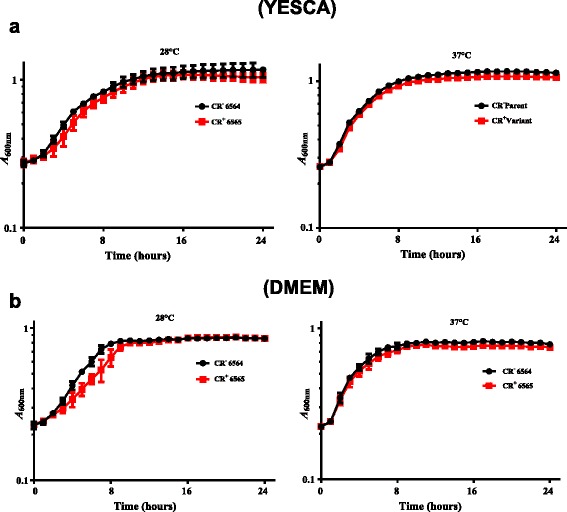
Comparison of bacterial growth characteristic in liquid cultures. Bacterial growth curves were generated by measuring optical density every 60 min of CR^−^ (NADC 6564) and the CR^+^ (NADC 6565) isolates grown in YESCA broth (**a**) and the minimal medium DMEM (**b**) at 28 °C and 37 °C for 24 h. Three independent cultures of each strain were grown in sets of three technical replicates and each time point on the growth curve represents an average of nine replicates. The growth rate was computed by taking into account the time (h) it took for optical density of the bacterial cultures to double during the exponential phase of the growth

Monitoring growth of the CR^+^ and CR^−^ isolates over a 24 h period in rich (YESCA broth) and minimal media (DMEM) revealed slightly reduced growth rate for the CR^+^ isolate at 28 °C and 37 °C (Fig. 2). For example, growth in YESCA broth at 28 °C resulted in doubling times of about 6.93 h and 6.18 h, respectively, for the CR^+^ and CR^−^ isolates (Fig. 2a). At 37 °C in YESCA broth, doubling times for the CR^+^ and the CR^−^ strains were 4.65 h and 4.68 h, respectively (Fig. 2a). In DMEM at 28 °C, doubling times for the CR^+^ and the CR^−^ strains were 5.92 h and 4.07 h, respectively (Fig. 2b). Growth in DMEM at 37 °C resulted in doubling times of about 3.62 h and 3.41 h for the CR^+^ and CR^−^ strains (Fig. 2b). Growth of both strains was slower at 28 °C as indicated by the longer doubling times compared to growth at 37 °C, irrespective of the media used. Overall, the CR^+^ isolate appeared to have slightly slower growth under the used growth conditions. The low growth rates (indicated by the very long doubling times) observed for both strains were due to the use of an automated, high-throughput system able to accommodate 300–400 μl of bacterial cultures per well of a 120-well plate. The magnitude of growth increases achieved in this system is very small thereby resulting in longer doubling times in comparison to the shorter doubling times that would normally be achieved when growth curves are performed in larger vessels amenable to agitation at higher speeds on a shaker.

### The transcriptome of CR^+^ isolate showed increased expression of biofilm-related genes but reduced expression of genes linked to stress responses

**Fig. 3:**
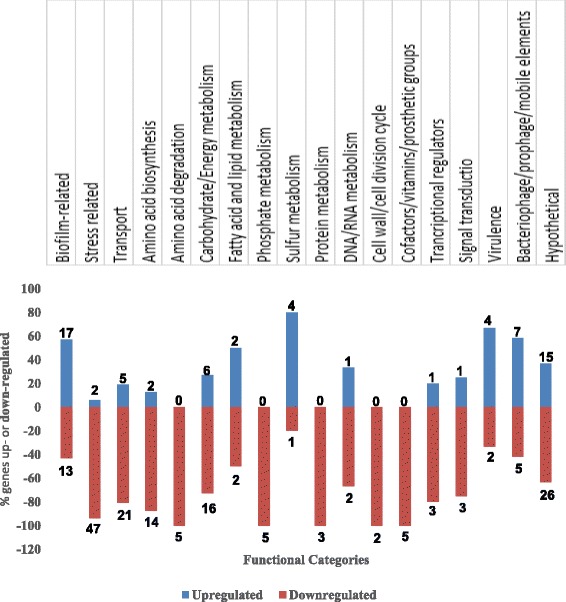
Graphic representation of differentially expressed genes in functional groups. Upregulated (*blue bars*) and downregulated genes (*red bars*) are shown as the percentage of total genes differentially expressed in each functional category. Only those genes that were expressed at ≥ 2.0-fold (*p* < 0.05) are used in this graphic display. The functional categories were selected from the RAST Server [44]

Although a total of 394 genes were differentially expressed in the CR^+^ strain (NADC 6565), only 242 genes showed significantly (*p* < 0.05) higher expression than the CR^−^ parental strain (NADC 6564). These genes were categorized into 18 functional categories based on the RAST subsystem predictions [44] and the percentage of genes up- or down-regulated in each category are shown in Fig. 3. As shown in Fig. 3, the majority of differentially expressed genes belonged to 6 of 18 functional classes. For example, the biofilm-related functional category contained 30 differentially expressed genes, of which 17 were up- and 13 were down-regulated (Fig. 3; Table 1). The upregulated genes promoting biofilm formation included *csgD, csgE*, *csgF* and *csgC*. Several genes belonging to the bacterial motility and chemotaxis regulon were also upregulated as these genes are linked to both the early and maturation stages of biofilm formation [47, 48]. Transcriptional regulators, such as QseB, FlhDC, YkgK, YdeH, Bdm, YdiV, which directly or indirectly modulate biofilm formation [49–53], were significantly altered in their expression in the CR^+^ isolate. Although ten genes related to cell envelope biosynthesis (Table 1) were downregulated in the CR^+^ isolate, seven of these (*ompA*, *ycfT*, *ygiB* and genes organized in the *yjbE, F, G, H* operon) have previously been shown to be differentially expressed in relation to biofilm formation [8, 54, 55]. Stress-related functional class contained the highest number of genes that were differentially expressed in the transcriptome of the CR^+^ isolate (Fig. 3 and Table 1). Of the 49 genes in this class, 47 were downregulated and controlled stress responses to acidity, oxidative metabolism, nutrient availability, cell envelope integrity, heat and osmolarity.

**Table 1:** List of differentially expressed genes mediating biofilm formation, cell envelope and global stress responses in Congo red-binding isolate of *E. coli* O157:H7

Gene group/gene^a^	Gene ID^b^	Known or predicted function^b^	Fold change	*P* value
Biofilm related				
Curli biosynthesis				
* csgF*	Z1671	Curli assembly protein	+2.40	0.03
* csgE*	Z1672	Curli assembly/transport	+2.14	0.09
* csgD*	Z1673	Positive regulator of curli genes	+2.07	0.05
* csgC*	Z1677	Autoagglutination protein	+5.70	0.06
Motility and chemotaxis				
* flgL*	Z1721	Flagellar hook-associated protein	+1.40	0.03
* cheR*	Z2938	Chemotaxi methyltransferase	+2.40	1.5E-04
* tap*	Z2939	Methyl-accepting protein IV	+4.25	6.6E-09
* tar*	Z2940	Methyl-accepting chemotaxis protein II	+4.48	1.7E-11
* cheA*	Z2942	Chemotaxis protein CheA	+2.82	1.8E-07
* motA*	Z2944	Flagellar motor protein MotA	+3.74	3.3E-08
* flhC*	Z2945	Transcriptional activator FlhC	+2.55	9.0E-04
* flhD*	Z2946	Transcriptional activator FlhD	+2.58	0.01
* fliZ*	Z3011	Flagellar biosynthesis protein FliZ	+1.57	0.05
* fliC*	Z3013	Flagellin	+2.54	2.4E-07
* fliD*	Z3014	Flagellar capping protein	+2.88	1.8E-07
* yfiR*	Z3897	Periplasmic protein	+1.55	0.03
Regulators				
* ykgK*	Z0361	Negative regulator of motility	−2.49	0.05
* bssR*	Z1062	Biofilm formation regulatory protein	+2.57	0.08
* ydeH*	Z2163	Putative diguanylate cyclase	−2.20	1.2E-05
* bdm*	Z2229	Biofilm-dependent modulation protein	−11.53	2.0E-07
* ydiV*	Z2736	Anti-FlhDC factor	+1.64	0.01
* qseB*	Z4377	Response regulator	−1.60	0.03
Cell envelope				
* nlpE (cutF*)	Z0204	Surface adhesion	−1.63	0.004
* ompA*	Z1307	Outer membrane protein	−2.50	8.6E-06
* ycfJ*	Z1749	Membrane protein	−24.98	1.5E-14
* ycfT*	Z1756	Inner membrane protein	−3.25	6.5E-04
* ygiB*	Z4394	Outer membrane protein	−1.55	0.01
* upp*P	Z4410	Undecaprenyl pyrophosphate phosphatase	−1.72	0.01
* yjbE*	Z5624	Predicted protein mediating extracellular polysaccharide biosynthesis	−19.61	1.0E-03
* yjbF*	Z5625	Predicted protein mediating extracellular polysaccharide biosynthesis	−14.61	3.0E-04
* yjbG*	Z5626	Predicted protein mediating extracellular polysaccharide biosynthesis	−12.87	2.0E-03
* yjbH*	Z5627	Predicted protein mediating extracellular polysaccharide biosynthesis	−7.24	2.1E-05
Stress related				
Acid stress				
* gadB*	Z2215	Glutamate decarboxylase	−14.41	3.0E-03
* xasA*	Z2216	Acid sensitivity protein GadC	−17.72	7.5E-05
* yhiM*	Z4890	Inner membrane protein	−14.43	0.01
* yhiD*	Z4920	Mg^2+^ transport ATPase	−12.48	0.01
* hdeD*	Z4923	Acid-resistance membrane protein	−13.47	5.0E-03
* gadA*	Z4930	Glutamate decarboxylase	−13.99	0.01
Oxidative stress				
* ybjW*	Z1107	Hydroxylamine reductase	+1.98	0.01
* marA*	Z2170	Transcriptional activator	−1.52	0.04
* marR*	Z2171	Transcriptional regulator	−1.56	0.05
* katE*	Z2761	Hydroxyperoxidase or catalase II	−3.18	6.7E-05
* cspD*	Z2117	Stationary phase/starvation-inducible	+1.65	0.02
* csiD*	Z3956	Carbon starvation-inducible protein	−5.85	3.1E-03
* yjiY*	Z5953	Carbon starvation protein	−5.74	7.0E-05
Cell envelope stress				
* ykfE*	Z0277	C-lysozyme inhibitor	−7.09	5.6E-06
* yccA*	Z1322	Putative carrier/transport protein	−1.54	4.0E-03
* ycfS* (*idtC*)	Z1752	L, D-transpeptidase	−2.73	5.5E-04
* spy*	Z2775	Protease/chaperone	−14.70	3.9E-09
* cpxP*	Z5458	Inhibitor of CpxA of CpxRA pathway	−3.76	3.5E-07
* slt*	Z5994	lytic murein transglycosylase	−1.54	0.01
Heat, osmotic and desiccation stress				
* htrA* (*degP*)	Z0173	Serine endoprotease	−2.39	0.01
* hslJ*	Z2330	Heat-shock outer membrane protein	−5.41	1.7E-11
* pspE*	Z2477	Thiosulfate:cyanide sulfur transferase	−2.23	9.3E-05
* pspD*	Z2478	Inner membrane phage shock protein	−3.38	6.9E-06
* pspC*	Z2479	Transcriptional activator	−3.42	3.2E-05
* pspB*	Z2480	Phage shock protein	−3.65	8.6E-06
* pspA*	Z2482	Phage shock protein	−3.29	2.0E-04
* pspF*	Z2484	Transcriptional regulator of phage shock proteins operon	−1.27	0.04
* htpX*	Z2876	Heat shock protein	−2.66	2.1E-05
* rpoH*	Z4835	Sigma factor σ^32^	−1.35	0.03
* rcsA*	Z3041	Positive regulator of colanic acid biosynthesis	−15.51	8.6E-04
* wzzB*	Z3189	Regulator of O-antigen component of LPS chains	−1.41	0.04
* wcaM*	Z3207	Colanic acid biosynthesis protein	−6.36	0.01
* wcaK*	Z3209	Pyruvyl transferase	−12.75	0.03
* cpsG*	Z3212	Phosphomannomutase	−14.59	3.4E-03
* cspB*	Z3213	Mannose-1-phosphate guanylly-trasferase	−13.91	7.0E-04
* wcaI*	Z3214	Glycosyl transferase	−14.28	4.0E-03
* wcaH*	Z3215	GDP-D-mannose dehydratase	−13.95	3.1E-03
* wcaG*	Z3216	Nucleotide di-P-sugar epimerase	−16.20	0.03
* gmd*	Z3217	GDP-D-mannose dehydratase	−15.73	0.04
* wcaF*	Z3218	Colanic acid biosynthesis acetyl-transferase	−12.03	0.01
* wcaE*	Z3219	Glycosyl transferase	−13.67	0.03
* wcaD*	Z3220	Colanic acid biosynthesis protein	−9.84	0.01
* wcaC*	Z3221	Glycosyl transferase	−8.14	9.6E-05
* wcaA*	Z3223	Glycosyl transferase	−16.40	1.1E-03
* wzc*	Z3224	Tyrosine kinase	−18.28	4.0E-03
* wzb*	Z3226	Tyrosine phosphatase	−18.15	0.01
* wza*	Z3227	Polysaccharide export protein	−19.40	9.0E-03
* osmY*	Z5977	Periplasmic chaperone	−2.44	0.02

^a^Gene group/gene designations were selected from RAST Server [44]

^b^Gene ID and known or predicted functions are based on the annotated sequence of *E. coli* O157:H7 EDL 933 [92]. Symbols + and – represent upregulated and downregulated gene expression, respectively

### The transcriptome of CR^+^ isolate revealed down regulation of specific transport systems and metabolic pathways

Growth in rich and minimal media resulted in slightly longer doubling times for the CR^+^ isolate at 28 °C and 37 °C (Fig. 2). The transcriptome of the CR^+^ isolate also revealed significantly reduced expression of genes encoding specific transport systems and pathways essential for amino acid, carbohydrate, energy, phosphate, protein and cofactor metabolism (Fig. 3 and Table 2). For example, of the 26 differentially expressed transport-related genes, 21 genes were down- and 5 genes were up-regulated in their expression. Several downregulated genes encoded transporters of ABC superfamily predicted to transport putrescine, glutamine, leucine/isoleucine/valine and dipeptides [56–59]. Other downregulated genes encoded outer membrane porins (phosphoporin, OmpA), permeases (N-acetylglucosamine PTS permease) and γ-amino butyrate transporter [60–63]. The genes that were upregulated in their expression encoded for a multidrug efflux transporter (*mdtH*), nitrite/nitrate antiporter (*nark*), permease for D-serine transport (*yhaO*), and biotin transporter (*yigM*) [64–67]. A number of key genes required for the biosynthesis and catabolism of amino acids showed reduced expression in the CR^+^ isolate (Fig. 3, Table 2). Only two genes, one encoding for lysine and the other encoding for aspartate biosynthesis showed higher expression in the mutant strain. Sixteen of the 22 genes implicated in carbohydrate/energy metabolism were downregulated in the CR^+^ isolate (Fig. 3, Table 2). Most other functional categories of genes, such as those involved in phosphate metabolism, cofactors and vitamin biosynthesis, fatty acid and lipid metabolism, sulfur metabolism, DNA/RNA/protein metabolism, transcriptional regulation and cell wall/cell division cycle, contained less than 10 genes and majority of these were downregulated in the CR^+^ isolate (Table 2 and Fig. 3). In the virulence genes functional category, four genes (*nleB*, *nleE, efa-1`*, Z4333) were up- and two (*cutF*, *ykfE*) were down-regulated (Fig. 3, Table 2). The latter two genes have been described in biofilm and stress-related functional categories. The *nleB* and *nleE* genes are effector proteins encoded by prophages and suggested to have virulence potential by suppressing the inflammatory response of the host [68, 69]. The *efa-*1gene along with the plasmid encoded *toxB* gene is implicated in the regulation of LEE-encoded virulence genes in *E. coli* O157:H7 [70]. However, the *efA-1`* and Z4333 virulence proteins are N-terminal and C-terminal homologs of the corresponding regions of a functional *efa-1* gene in non-O157:H7 *E. coli* strains [70].

**Table 2:** List of differentially expressed genes showing altered metabolic profile of *E. coli* O157:H7 Congo red-positive isolate

Gene group/gene^a^	Gene ID^b^	Known or predicted function^b^	Fold change	*P* value
Transport				
* phoE*	Z0302	Outer membrane phosphoporin protein	−1.89	0.027
* ybaL*	Z0597	Cation:proton antiport protein	−1.48	0.004
* ybbA*	Z0648	ABC transporter ATP-binding protein	−1.59	5.4E-04
* ybbP*	Z0649	Oxidoreductase	−1.7	0.02
* nagE*	Z0826	N-acetylglucosamine PTS permease	−2.30	0.022
* glnQ*	Z1031	Glutamine ABC transporter ATP-binding	−3.09	0.006
* glnH*	Z1033	Glutamine ABC transporter periplasmic protein	−3.66	0.004
* potF*	Z1081	Putrescine ABC transporter periplasmic-binding protein	−4.42	4.0E-04
* potG*	Z1082	Putrescine ABC transporter ATP-binding protein	−4.00	9.0E-04
* potH*	Z1083	Putrescine ABC transporter membrane protein	−2.98	1.5E-02
* potI*	Z1084	Putrescine ABC transporter membrane	−2.92	9.0E-03
* lolA*	Z1237	Periplasmic chaperone for translocation of lipoproteins to outer membrane	−1.48	0.037
* ompA*	Z1307	Outer membrane porin A	−2.50	8.6E-06
* ybaL*	Z1672	Cation:proton antiporter	−1.48	0.004
* VybbA*	Z1673	ATP-binding protein of ABC transporter	−1.59	5.0E-04
* ybbP*	Z1677	Membrane component of ABC transporter protein	−1.59	5.0E-04
* yceL* (*mdtH*)	Z1702	Multidrug efflux transporter	+1.40	0.024
* narK*	Z2000	Nitrite/nitrate antiporter	+2.79	0.059
* gabP*	Z3961	Gamma-aminobutyrate transporter	−5.33	1.0E-03
* yhaO*	Z4463	Permease for D-serine transport	+2.76	0.01
* livF*	Z4824	Leucine/isoleucine/valine transporter ATP-binding subunit	−2.06	0.04
* nirC*	Z4728	Transport of nitrite	+3.95	0.03
* dppF*	Z4957	Dipeptide transporter ABC-binding unit	−2.61	0.03
* dppD*	Z4958	Dipeptide transporter ABC-binding unit	−2.40	0.04
* dppA*	Z4961	Dipeptide transporter protein	−2.52	0.03
* yigM*	Z5348	Biotin transporter	+1.36	0.03
Amino acid biosynthesis				
* thrB*	Z0003	Homoserine kinase	−2.18	0.01
* thrC*	Z0004	Threonine synthase	−2.17	0.03
* leuD*	Z0080	Isopropylmalate isomerase small subunit	−2.66	0.01
* leuC*	Z0081	Isopropylmalate isomerase large subunt	−2.34	0.04
* asnB*	Z0821	Asparagine synthetase B	−3.07	0.049
	Z2491	Glutamine synthetase	−2.93	0.02
* hisC*	Z3183	Histidinol-phosphate aminotransferase	−1.79	0.03
* hisH*	Z3185	Imidazolr glycerol phosphate synthase	−1.78	0.03
* hisA*	Z3186	Imidazole-4-carboxamide isomerase	−1.84	0.02
* hisF*	Z3187	Imidazole glycerol phosphate synthase	−1.86	0.01
* hisI*	Z3188	Bifunctional phosphoribosyl-AMP cyclohydrolase/phosphoribosyl-ATP pyrophosphatase	−1.82	0.02
* lysA*	Z4156	Diaminopimelate decarboxylase	+2.39	0.03
* ilvN*	Z5164	Acetolactate synthase I regulatory subunit	−2.08	0.02
* ilvB*	Z5165	Isoleucine/valine biosynthesis	−2.02	0.01
* asnA*	Z5245	Asparagine synthetase	−3.47	0.04
* aspA*	Z5744	Aspartate ammonia-lyase	+2.04	0.04
Amino acid degradation				
* goaG*	Z2486	4-aminobutyrate transferase	−6.75	2.5E-04
* ordL*	Z2487	γ-glutamylputrescine oxidase	−6.79	0.01
* aldH*	Z2488	γ-glutamyl-γ-aminobutyraldehyde	−5.52	1.1E-03
* gabD*	Z3959	Succinate-semialdehyde dehydrogenase I	−4.12	5.0E-03
* gabT*	Z3960	4-aminobutyrate aminotransferase	−5.66	5.0E-03
Carbohydrate/Energy metabolism				
* aceF*	Z0125	Dihydrolipoamide acetyltransferase	−2.00	0.02
* yaeM*	Z0184	1-deoxy-D-xylulose 5-phosphate reductoisomerase	−1.33	0.03
* malZ*	Z0501	Maltodextrin glucosidase	−2.13	0.041
* ybdR*	Z0752	Zn-dependent oxidoreductase	−2.70	3.0E-04
* sdhA*	Z0877	Succinate dehydrogenase flavoprotein subunit	−3.11	0.044
* sucA*	Z0880	2-oxoglutarate dehydrogenase E1	−2.73	0.047
* sucB*	Z0881	Dihydrolipoamide succinyltransferase	−2.62	0.039
* sucC*	Z0882	Succinyl-CoA synthetase subunit beta	−2.64	0.029
* sucD*	Z0883	Succinyl-CoA synthetase subunit alpha	−2.37	0.054
* nadA*	Z0919	Quinolate synthetase	−1.82	0.002
* galM*	Z0926	Galactose-1-epimerase	−1.33	0.047
* galE*	Z0929	UDP-galactose-4-epimerase	−1.46	0.019
* ybjW*	Z1107	Hydroxylamine reductase	+1.98	9/0E-03
* galU*	Z2012	UTP-glucose-1-phosphate uridylyltransferase	−2.11	0.006
* adhE*	Z2016	Aldehyde-alcohol dehydrogenase	+2.29	0.01
* manX*	Z2860	PTS system mannose-specific transporter unit AB	+1.47	0.035
* manY*	Z2861	PTS enzyme IIC, mannose-specific	+1.55	0.007
* manZ*	Z2862	PTS system mannose-specific transporter subunit IID	+1.50	0.02
* yghA*	Z4356	Oxidoreductase	−2.72	0.01
* ugpB*	Z4822	Glycerol-3-phosphate transporter periplasmic-binding protein	−2.74	0.01
* uhpT*	Z5156	Sugar phosphate antiporter	+3.22	6.9E-05
* mdoB*	Z5959	Phosphoglycerol transferase I	−3.00	3.0E-04
Fatty acid and lipid metabolism				
* pgsA*	Z3000	Phosphatidylglycerophosphate synthetase	+1.47	0.01
* plsC*	Z4372	1-acyl-sn-glycerol-3-phosphate	+1.30	2.3E-03
* yrbB*	Z4554	Phospholipid ABC transporter	−1.87	2.0E-03
* yrbC*	Z4555	Phospholipid ABC transporter	−1.97	3.0E-04
Phosphate metabolism				
* phoU*	Z5215	Transcriptional regulator	−2.09	4.0E-03
* pstB*	Z5216	Phosphate transporter ATP-binding protein	−2.02	0.02
* pstA*	Z5217	Phosphate transport permease subunit PstA	−2.70	2.0E-03
* pstC*	Z5218	Phosphate transport permease subunit PstC	−2.52	3.0E-04
* pstS*	Z5219	Phosphate ABC transporter substrate-binding protein	−5.84	1.4E-07
Sulfur metabolism				
* cysZ*	Z3679	Sulfate transport protein	−1.47	0.03
* cysA*	Z3687	Sulfate/thiosulfate transporter	+1.50	0.02
* cysW*	Z3688	Sulfate/thiosulfate permease	+2.16	3.4E-03
* cysN*	Z4059	Sulfate adenylyltransferase subunit 1	+1.65	0.03
* cysJ*	Z4074	Sulfite reductase subunit alpha	+2.12	1.2E-03
Protein metabolism				
* yaeJ*	Z0203	Peptidyl-tRNA hydrolase domain-containing protein	−1.36	0.03
* rpmE*	Z5484	50S ribosomal protein L31	−2.20	0.019
* pepA*	Z5872	Leucyl aminopeptidase	−1.58	0.02
DNA/RNA metabolism				
* hisT*	Z3580	tRNA pseudouridine synthase A	−1.51	0.02
* deoD*	Z5986	Purine nucleoside phosphorylase	−1.37	0.04
* mazG*	Z4096	Nucleoside triphosphate pyrophospho-hydrolase	+1.49	0.02
Cell wall/cell division/cell cycle				
* murD*	Z0098	UDP-N-acetylmuramoyl-L-alanyl-D-glutamate synthetase	−1.58	0.02
* uppP*	Z4410	Undecaprenyl pyrophosphate phosphatase	−1.72	0.01
Cofactors, vitamins, protheticgroups				
* yaeM*	Z0184	1-deoxy-D-xylulose 5-phosphate	−1.33	0.04
* nadA*	Z0919	Quinolinate synthetase reductoisomerase	−1.82	0.002
* btuR*	Z2540	Cobinamide adenosyltransferase	−1.31	0.04
* nadB*	Z3856	L-aspartate oxidase	−1.63	4.0E-03
* ggt*	Z4813	Gamma-glutamyltranspeptidase	−1.76	2.0E-03
Transcriptional regulation				
* ykgK*	Z0361	LuxR-type transcriptional regulator	−2.49	0.049
* marA*	Z2170	DNA-binding transcriptional activator MarA	−1.52	0.039
* marR*	Z2172	MarR family transcriptional repressor	−1.56	0.053
* yhgG*	Z4765	Transcriptional regulator	+2.12	0.04
Signal transduction				
* phoB*	Z0497	Response regulator belonging to PhoB/PhoR two-component system	−1.53	0.031
* phoR*	Z0498	Signal sensor component of PhoB/PhoR two-component system	−1.47	0.036
* narL*	Z1996	Nitrate/citrate response regulator	−1.48	0.064
* narK*	Z2000	Nitrite/nitrate antiporter	+2.79	0.059
Virulence				
* cutF* (*nlpE*)	Z0204	Lipoprotein involved with copper homeostasis and adhesion	−1.63	0.004
* ykfE*	Z0277	C-lysozyme inhibitor	−7.09	5.6E-06
* nleB*	Z4328	Type III effector	+1.72	0.04
* nleE*	Z4329	Type III effector	+1.86	0.02
* efa-1`*	Z4332	Cytotoxin	+1.84	0.01
	Z4333	Cytotoxin	+1.93	0.03

^a^Gene group/gene designations were selected from RAST Server [44]

^b^Gene ID and known or predicted functions are based on the annotated sequence of *E. coli* O157:H7 EDL 933 [92]. Symbols + and – represent upregulated and downregulated gene expression, respectively

Twelve genes that were differentially expressed (7 up- and 5 down-regulated) in the CR^+^ isolate were assigned to the bacteriophage/prophage/mobile element category (Table 3, Fig. 3). Eight of these genes were hypothetical proteins encoded by phage CP-933 and others encoded a putative cI repressor (Z0309), integrase (Z2036) and a regulator (Z2970). In the second largest category containing 41 differentially expressed genes, 33 genes were assigned hypothetical and 8 genes putative functions. Large majority (26 genes) of these genes were downregulated in the CR^+^ isolate (Table 3, Fig. 3). The transcriptomes of the strains 6564 and 6565 have been deposited at the GenBank under the following accession numbers (SRR4436361 for the strain 6564; SRR4436642 for the strain 6565).

**Table 3:** List of differentially expressed genes related to bacteriophages, mobile elements and hypothetical functions in *E. coli* O157:H7 Congo red-positive isolate

Gene group/gene^a^	Gene ID^b^	Known or predicted function^b^	Fold change	*P* value
Bacteriophage related				
	Z0309	Putative cI repressor protein for prophage CP-933H	+1.43	0.03
	Z1460	Hypothetical protein encoded by bacteriophage Bp-933 W	−4.12	0.01
	Z1922	Hypothetical protein encoded by bacteriophage CP-933X	−2.23	−0.03
	Z1923	Hypothetical protein encoded by bacteriophage CP-933X	−2.38	−0.04
* intO*	Z2036	Integrase for bacteriophage CP-933O	−1.26	0.01
* coxT*	Z2970	Regulator for prophage CP-933 T	+4.42	0.04
	Z2971	Phage CP-933 T encoded hypothetical protein	+3.48	0.03
	Z2972	Phage CP-933 T encoded hypothetical protein	+3.70	0.01
	Z2973	Phage CP-933 T encoded hypothetical protein	+4.10	0.01
	Z2974	Phage CP-933 T encoded hypothetical protein	+2.83	0.04
	Z3305	Bacteriophage CP-933 V encoded protein	−1.62	0.01
	Z4330	Transposase	+1.94	0.03
Hypothetical				
	Z0005	Unknown	−7.49	4.7E-08
* yagU*	Z0353	Putative acid resistance	2.29	0.004
* yagY*	Z0359	Predicted pilus chaperone (cryptic)	−2.13	0.04
* yagZ*	Z0360	Predicted pilus major subunit	−2.83	0.001
* yaiY*	Z0475	Predicted inner membrane protein	−33.49	1.6E-12
* yaiE*	Z0487	Unknown	−1.49	0.01
* yajI*	Z0513	Unknown	−3.71	1.3E-06
* ybcI*	Z0682	Predicted inner membrane protein	−2.48	0.004
	Z0879	Unknown	−2.95	0.04
* ybjO*	Z1085	Predicted inner membrane protein	−2.35	4.3E-04
* ybjX*	Z1112	Unknown	−1.51	0.01
* ymcD*	Z1404	Unknown	+1.48	3.7E-03
	Z1528	Unknown	+2.11	0.03
* yceI*	Z1692	Unknown	−2.24	5.0E-03
* yceB*	Z1700	Unknown	−1.89	5.0E-04
* ycfJ*	Z1749	Unknown	−24.98	1.5E-14
	Z2283	Unknown	−4.39	3.0#-06
* ydeH*	Z2292	Unknown	+2.69	2.0E-04
* ydeF*	Z2308	Unknown	+2.83	2.7E-06
	Z2421	Unknown	+1.79	0.03
* yoaG*	Z2838	Unknown	+2.54	0.02
	Z2839	Unknown	+2.36	0.04
	Z2893	Unknown	−1.61	0.01
* rcnB*	Z3275	Putative nickel/cobalt efflux protein	−1.61	0.02
* ypeC*	Z3656	Unknown	−23.74	1.9E-17
* ypfG*	Z3722	Unknown	−4.58	9.0E-09
	Z3965	Unknown	−2.33	1.5E-03
	Z4126	Unknown	−4.31	3.0E-04
	Z4151	Unknown	−3.84	7.4E-07
	Z4267	Unknown	+5.52	2.3E-03
	Z4268	Unknown	+4.92	4.3E-06
* yggG*	Z4280	Unknown	−1.71	0.02
	Z4318	Unknown	+1.30	0.04
* ygjT*	Z4441	Unknown	−2.25	5.0E-03
* yhaM*	Z4462	Unknown	+2.34	0.03
* yiaB*	Z4988	Unknown	−4.71	5.0E-03
* yiiF*	Z5432	Unknown	+1.52	0.04
* yjcO*	Z5677	Unknown	+1.28	0.04
	Z5694	Unknown	−12.64	6.7E-09
* yjdA*	Z5711	Putative DNA replication function	+2.23	6.4E-06
* yjcJ*	Z5712	Unknown	+2.79	9.4E-05
* yjfN*	Z5795	Unknown	−2.58	8.0E-04

^a^Gene group/gene designations were selected from RAST Server [44]

^b^Gene ID and known or predicted functions are based on the annotated sequence of *E. coli* O157:H7 EDL 933 [92]. Symbols + and – represent upregulated and downregulated gene expression, respectively

### Phenotypic assays validated the transcriptomic data

**Fig. 4:**
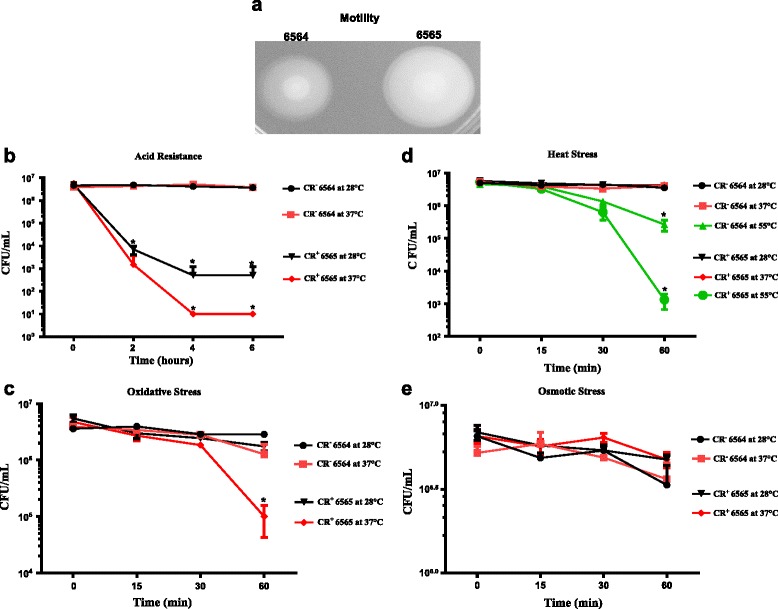
Validation of the transcriptomic data by phenotypic assays. Bacterial cultures were grown overnight in LB-No Salt broth at 28 °C on a shaker (170 rpm). For motility assays, 2 μl of undiluted cultures were spotted on the surface of a soft motility agar plate (**a**). For other assays, overnight cultures were diluted appropriately into a suitable medium, and incubated at desired temperatures as described under Methods. Aliquots (100 μl) were withdrawn at 0, 2, 4 and 6 h intervals for measuring acid resistance (**b**) or at 0, 15, 30 and 60 min intervals for measuring tolerance to oxidative (**c**), heat (**d**) and osmotic (**e**) stressors. Each *time point* represents means of three independent assays and *error bars* represent standard deviation of 2. The *time point* with an * above them indicates *p* < 0.05 when the mutant was compared to the parental strain

Several phenotypic assays were performed to verify that differential expression of genes with known functions correlated with their predicted contributions to the expression of the tested phenotype in the CR^+^ isolate relative to the CR^−^ parental strain. As shown in Fig. 1, Congo red binding, biofilm formation and curli production phenotypes were some of the attributes that distinguished CR^+^ isolate from the CR^−^ parental strain. The expression of genes, especially *csgD*, *csgE* and *csgF* of *csgDEFG* and *csgC* of *csgBAC* operons (Table 1), which are essential for curli biosynthesis, was significantly higher in the CR^+^ isolate. The significantly lower expression of *qseB* and *ompA*, the two genes known to affect biofilm formation [52, 55] and that are assigned to the biofilm-related functional category (Table 1), was also in agreement with enhanced biofilm formation in the CR^+^ isolate. We also examined the motility phenotype because expression of several genes that encode proteins for swimming motility, chemotactic functions and contribute to initial and final stages of biofilm formation [47, 48] were significantly upregulated in the CR^+^ mutant strain (Table 1). As shown in Fig. 4a, the CR^+^ isolate (NADC 6565) produced larger motility zones on soft motility agar plates at 37 °C compared to the CR^−^ parental strain (NADC 6564). In the stress-related functional category, several genes (*gadA, gadB* and *xasA* or *gadC*) encoding decarboxylases and glutamic acid:γ-aminobutyrate antiporter of the acid resistance pathway 2 expressed at much lower levels in the CR^+^ isolate compared to the CR^−^parental strain (Table 1). When these two strains were compared for their survival post-exposure to pH 2.5, the survival of the CR^+^ isolate was reduced by 2–3 logs after 2 to 4 h of exposure at 28 °C to this pH (Fig. 4b). The survival rate of the CR^+^ isolate was strongly affected (reduction by 3–5 logs) at 37 °C (Fig. 4b). We also compared the survival response of the CR^+^ isolate and the CR^−^ parental strain to oxidative stress since some of the genes (*katE* and *ahpC*) that provide protective functions against this type of stress were downregulated in CR^+^ isolate (Table 1). As shown in Fig. 4c, the survival of the CR^+^ isolate was reduced by about 2 logs after 60 min exposure at 37 °C (Fig. 4c). As shown in Table 1, transcriptional levels of several genes (*htrA*, *htpX* and *hslJ*) encoding heat shock proteins, heat shock σ32, phage shock proteins and proteins mediating colanic acid biosynthesis were downregulated in the CR^+^ strain. Since many of these proteins (HtrA, HtpX and RpoH) are direct contributors to mitigation of the cellular damage incurred in response to exposure of bacterial cells to temperatures above 42 °C [71–73], we compared survival of the CR^+^ isolate to the CR^−^ parental strain by exposing bacterial cells to a range of temperatures. Significantly higher reductions (> 3 logs) in cell viability were observed for the CR^+^ isolate at 55 °C relative to the parental strain (1 log) (Fig. 4d). It has been well documented that one stress could provide cross-protection against other stresses [74, 75]. Carbon starvation stress response, which is mediated by σ^S^, can induce cross-protection to many different stresses, including osmotic stress [75]. Carbon source starvation stress response induces the expression of carbon starvation as well as stationary phase/starvation proteins. As shown in Table 1, transcription of two carbon starvation genes (*csiD* and *yjiY*) and many genes involved in acid, oxidative, heat and desiccation resistance was downregulated in the CR^+^ isolate. Therefore, we examined the osmotic shock resistance by exposing this strain to a high external osmotic environment. As shown in Fig. 4e, no significant differences were observed in tolerance to high osmotic stress between the CR^+^ isolate and the CR^−^ parental strain at both 28 °C and 37 °C.

### The CR^+^ phenotype correlated with the disruption of the *rcsB* gene by a 5-bp tandemly duplicated sequence

The reported mechanisms for the expression of the CR^+^ phenotype in otherwise CR^−^ strains of *E. coli* O157:H7 have either been the promoter mutations causing upregulation of *csgD* expression or the mutations in genes, such as *rpoS, mlrA* and *rcsB*, encoding negative transcriptional regulators of *csgD* [17, 36, 76, 77]. For example, disruption of *mlrA* with a Shiga-toxin encoding bacteriophage and intragenic deletions of the *rcsB* gene caused by the presence of insertion-sequence (IS) elements have been linked to the inability or the ability to produce biofilms, respectively, in various strains of *E. coli* O157:H7 [17, 77]. Similarly, sequence heterogeneity in the *rpoS* gene causing single amino acid substitutions or premature protein truncations has been linked to variability in Congo red binding and biofilm production [77]. Preliminary analysis of the *csgD* promoter sequence, determination of the presence of a functional RpoS by a catalase test, and confirmation of the presence of an intact *rcsB* by PCR revealed similar results (presence of a wild-type *csgD* promoter sequence, functional RpoS and intact *rcsB* gene) for both the CR^+^ mutant (NADC 6565) and CR^−^ (NADC 6564) parental strains (data not shown). In order to determine the genetic basis of the CR^+^ phenotype and biofilm-producing ability of the strain NADC 6565, we compared the genome sequence of the NADC 6565 to that of NADC 6564, which are available at the GenBank under the accession numbers CP017251 and CP017252 for NADC 6564 and CP017249 and CP017250 for NADC 6565. The genome comparison revealed identical *rpoS* and *csgD* promoter sequences except that the *rcsB* gene contained a tandem duplication of a 5-bp sequence CAGTG in the CR^+^ isolate NADC 6565. The alignment of the nucleotide sequences of the *rcsB* genes of the CR^+^ mutant and CR^−^ parental isolates showed the insertion of an extra 5-bp sequence (CAGTG) at nucleotide 423 resulting in the disruption of the *rcsB* ORF past nucleotide 429 in the CR^+^ isolate. This disruption in the *rcsB* ORF resulted in the truncation of the RcsB polypeptide at amino acid 144 in the CR^+^ mutant isolate compared to the 216-amino acid, full length RcsB polypeptide produced in the CR^−^ parental strain.

### Complementation with the *rcsB* gene of the CR^−^ parental strain restored parental phenotypes on the CR^+^ isolate

**Fig. 5:**
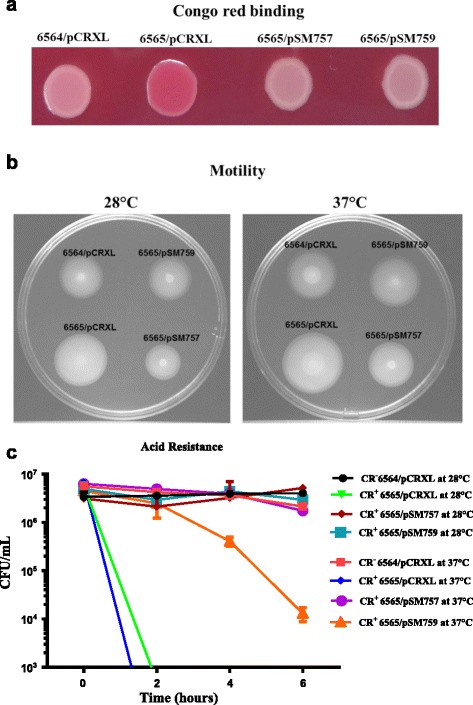
Determination of the ability of the *rcsB* gene of the CR^−^ parental strain (NADC 6564) for complementing the CR^+^ isolate (NADC 6565) for the phenotypes putatively altered due to the loss of the RcsB function. **a** The overnight cultures of the CR^−^ parental strain carrying the empty vector pCRXL and the CR^+^ mutant strain complemented with plasmid pSM757 (pCRXL carrying a cloned copy of the *rcsDB* operon), pSM759 (pCRXL carrying a cloned copy of the *rcsB* gene), or pCRXL were grown at 28 °C in LB-No Salt broth. Aliquots (5-μl) of these cultures were spot-inoculated on Congo red-containing agar plates. After 48 h of incubation at 28 °C, the color (*white* or *red*) of the growth produced at the spots of inoculation was photographed. **b** The overnight cultures of the CR^−^ parental strain carrying pCRXL and the CR^+^ mutant strain complemented with plasmid pSM757, pSM759 or pCRXL were grown at 37 °C in LB broth and 2-μl aliquots of these cultures were spot-inoculated on soft-motility agar plates. After incubation at 28 °C or 37 °C for appropriate length of time, the motility zones (visible as *white-colored rings* at the spot of inoculation) produced on these plates were captured by photographing. **c** The CR^−^ parental strain carrying the pCRXL vector and the CR^+^ mutant strain complemented with plasmid pSM757 (pCRXL carrying a cloned copy of the *rcsDB* operon), pSM759 (pCRXL carrying a cloned copy of the *rcsB* gene), or pCRXL were grown (LB-No Salt, pH 5.5) overnight at 28 °C. These cultures were diluted 1:1000 in LB No Salt (pH 2.5) and incubated at 28 °C or 37 °C. Aliquots (100 μl) were withdrawn from these cultures at 0, 2, 4 and 6 h intervals and 10-fold serial dilutions of these cultures were plated on LB-agar plates containing kanamycin. After incubation at 37 °C, the numbers of colonies produced on these plates were counted (CFU/ml) and plotted against time (h) on a XY graph. Each *time point* represents means of three independent assays and *error bars* represent standard deviation of 2

Since transcriptome of the CR^+^ isolate (NADC 6565) showed increased expression of genes required for curli biosynthesis and swimming motility but reduced expression of genes required for acid resistance, we determined if *in trans* complementation of the CR^+^ isolate with recombinant plasmids carrying either the *rcsB* gene or the *rcsDB* operon of the CR^−^ parental isolate (NADC 6564) would change Congo red binding, swimming motility and acid resistance phenotypes of the CR^+^ isolate similar to those produced by the CR^−^ parental isolate. The CR^+^ isolate containing the empty cloning vector (pCRXL) produced red bacterial growth compared to the white growth of the CR^−^ parent containing pCRXL (Fig. 5a). When complemented with the recombinant plasmid pSM757 (pCRXL::*rcsDB*) or pSM759 (pCRXL::*rcsB*), the CR^+^ isolate produced white growth similar to that of the CR^−^ parent containing pCRXL (Fig. 5a). Similarly, the CR^+^ isolate complemented with pCRXL produced motility zones larger in size compared to the parent strain carrying pCRXL at both 28 °C and 37 °C, but when complemented with pSM757 or pSM759 the sizes of the motility zones produced were similar to or slightly smaller than that of the CR^−^ parental strain containing pCRXL (Fig. 5b). As shown in Fig. 5c, acid resistance of the CR^+^ isolate containing pCRXL was much lower than the CR^−^ parent containing pCRXL at both 28 °C and 37 °C as indicated by significantly (*p* < 0.05) lower (> 3 logs) recovery of viable cells of the CR^+^ isolate at both temperatures after 2 h exposure to very low pH (Fig. 5c). As is evident from Fig. 5, complementation with pSM757, which carries both *rcsD* and *rcsB* genes, was more effective in restoring Congo red binding, motility and acid resistance phenotypes on the CR^+^ mutant to the levels similar to those expressed in the CR^−^ parental strain compared to those by pSM759 encoding only *rcsB*. Because histidine kinase activity of RcsD is essential for phosphorylation of the response regulator RcsB [15, 18], the above results suggests that the suboptimal phosphorylation of RcsB due to the lack of histidine kinase activity-encoding *rcsD* on pSM759 might be responsible for only the partial restoration of the above phenotypes on the CR^+^ isolate.

## Discussion

We show here that *E. coli* O157:H7 strain 86–24, originally linked to a foodborne disease outbreak in the western USA in 1986, does not bind Congo red (CR^−^) but can give rise to Congo red binding isolates (CR^+^). The CR^+^ phenotype correlated positively with biofilm formation and presence of curli fimbriae at bacterial cell surfaces. Comparative transcriptomics revealed that majority (175/242) of the differentially expressed genes in the CR^+^ isolate were downregulated. The genes upregulated in expression accounted for only 28% (67/242) of the differentially expressed genes, and a large number of these genes were involved in pathways promoting biosynthesis of cell surface structures, such as curli fimbriae and flagella. The presence of abundant curli fimbriae at bacterial cell surfaces of the CR^+^ mutant isolate correlated with increased Congo red binding and biofilm formation, two phenotypes that are indicative of the increased expression of genes necessary for the biosynthesis of curli fimbriae [10]. Although, we detected ˃ 2-fold increases in the expression of genes *csgD, E, F* and *csgC* representing the two curli encoding operons *csgDEFG* and *csgBAC*, respectively, differential expression of *csgB* and *csgA* genes was not detected in the CR^+^ isolate but the expression of the *csgC* gene was the highest (5.7-fold). These lower than expected increases in the expression of some of the curli encoding genes could be attributed to the use of a minimal medium containing 0.1% glucose, which is not considered inhibitory to curli gene expression [37], and harvesting of bacterial cells as they reached early stationary phase (about 5 h of growth) for RNA preparation. An earlier study reported very large increases in the expression of *csgDEFG* and *csgBAC* operons using microarray analysis of RNA prepared from bacterial cultures grown in LB-no salt broth overnight at 28 °C [17]. However, differential expression of several other genes were found to be similar between the results presented here and the earlier study [17], indicating that older cultures (grown at 28 °C for 24 h) might be necessary for detecting higher levels of expression of curli encoding genes. The *csgC* gene encodes an anti-amyloid protein that prevents premature assembly of curlin protein during its passage through cell’s interior compartments to the bacterial cell surface [78]. Thus, it is possible that the prevention of premature assembly of curlin by increased levels of CsgC coupled with increased expression of assembly gene *csgF* might allow increased expression of curli fimbriae in the CR^+^ isolate despite no increases in the expression of *csgA*. Additional evidence supporting this assumption is the detection of abundant curli fimbriae on bacterial cells of the CR^+^ isolate compared to those of the CR^−^ parental strain.

Several genes encoding motility and chemotactic functions were also upregulated in the transcriptome of the CR^+^ isolate, the finding similar to those reported in an earlier study that used overnight grown bacterial cultures [17]. The increased expression of flagellar genes correlated with the downregulation of the expression of *bdm*, *ykgK* and *qseB* that are negative regulators of flagellar biosynthesis [50, 52, 79]. Multiple studies have shown that swimming motility is directly correlated to biofilm formation as it promotes bacterial swimming in liquid and viscous media enabling bacterial cells to reach surfaces of abiotic or biotic objects for final, reversible adherence mediated through adhesive structures, such as curli fimbriae [47, 48, 50, 79]. Interestingly, the expression of *ydeH*, which encodes a Zn-dependent diguanylate cyclase and represses bacterial motility and enhances biofilm formation through curli biosynthesis [80], was significantly downregulated in the transcriptome of CR^+^ isolate [81]. Thus, based on this data, *ydeH* might regulate flagellar gene expression, but it might be dispensable for curli gene regulation in the CR^+^ isolate. Although, we observed increased expression of *ydiV*, which is an EAL-domain type protein and inhibits flagellar production by inhibiting FlhDC, it is enzymatically inactive in *E. coli* [82]. Another deviate finding was the upregulation (2.6 fold) of biofilm regulator BssR, which has been shown to inhibit bacterial motility and biofilm formation when bacterial cells are grown in rich medium with glucose [83]. However, in the current study, *bssR* upregulation did not correlate with increased motility and biofilm formation in the CR^+^ isolate, either because a minimal media with 0.1% glucose was used in the current study or downregulation of other transcriptional regulators contributed to the observed phenotypes.

The majority of downregulated genes were associated with carbohydrate and energy metabolism, biosynthesis of amino acids, transport across cell membrane and stress response pathways. The downregulated genes were spread across all the major stress response pathways that are necessary for bacterial survival in environments with very low pH, high temperatures, nutritional limitations and for mitigating damaging effects of aerobic metabolism by quenching peroxides and superoxide byproducts. Most stress response pathways, such as CpxRA (envelope and high pH stresses), EnvZ/OmpR (external osmolarity) and RcsBCD (desiccation, osmolarity and complex signals) in *E. coli* consist of a membrane-anchored signal sensor and a cytoplasmic response regulator for affecting gene expression [14, 16, 20]. While none of the genes encoding these signal transduction pathways were differentially expressed in the transcriptome of the CR^+^ isolate, expression of many genes that respond to induction of these signaling systems were significantly reduced compared to the CR^−^ parental isolate. The downregulated genes were those necessary for coping with exposure to very low pH, heat, osmotic, desiccation, oxidative, envelope and nutritional stresses. Most of these downregulated genes encoded enzymes and/or cell membrane proteins to mitigate the effects of a specific stress with or without differentially expressed transcriptional regulators linked to the expression of these genes. For example, the downregulated genes for the acid resistance included *gadA* and *gadB*, encoding two almost identical glutamate decarboxylases, and several other genes (*yhiM*, *yhiD* and *hdeD*) encoding membrane anchored proteins needed for protection at very low pH exposures [84, 85]. This suggests that a differentially expressed transcriptional regulator or lack thereof in the CR^+^ isolate might be responsible for the down-regulation of these acid resistance genes by directly or indirectly affecting the expression of transcriptional regulators, such as GadE, GadX or GadW, which regulate acid resistance pathways in O157 [86].

Other downregulated genes identified in the transcriptome of the CR^+^ isolate were *ahpC* and *katE*, which encode hydroxyperoxidases as a part of the oxidative stress response protecting bacterial cells from the negative effects of hydrogen peroxide produced as a byproduct of aerobic metabolism [87]. While *ahpC* expression is OxyR-dependent and induced by sulfate starvation, the expression of *katE* is RpoS-dependent and induced by hyperosmotic stress and nutritional starvation [88]. Thus, it is possible that the increased expression of sulfate metabolism genes observed in the transcriptome of the CR^+^ isolate could have contributed to reduced expression of *ahpC*. On the other hand, downregulation of *katE* might have been due to the downregulation of genes impacted in heat shock and osmotic stress responses in the CR^+^ isolate. However, phenotypic assays showed no difference in the viability of the CR^+^ isolate relative to the CR^−^ parental strain under hyperosmotic conditions, while the survival of the CR^+^ isolate in the presence of H_2_O_2_ was significantly lower at 37 °C compared to 28 °C, suggesting that *katE* expression might be correlated to the heat shock response. Two sigma factors, RpoH (σ^32^) and RpoE (σ^24^), control expression of many genes that are induced in response to heat shock [23, 25]. The RpoH-activated heat shock genes are turned on when cells are grown at 30–43 °C, and temperatures above 45 °C result in the synthesis of mostly the RpoE-activated heat shock proteins. The downregulated heat shock genes in the transcriptome of CR^+^ isolate included RpoH-dependent *htpX*, and RpoE-dependent *htrA* (*degP)* and σ^32^. The heat shock assays showed very poor survival of the CR^+^ mutant at 55 °C compared to the parental strain indicating that the reduced expression of *rpoH* at high temperatures prevents the induction of heat shock response. Since *rpoE* was not differentially expressed in the CR^+^ isolate, the downregulation of *htrA* implies the role of *rpoH* or another regulator that was downregulated or lacking in this isolate.

We also observed downregulation of genes in the CR^+^ isolate that are associated with nutritional, envelope, heat and osmotic stresses but that are not regulated by the heat shock sigma factors. Prominent among these were the entire *wca* gene cluster, which encodes genes mediating colanic acid biosynthesis and a positive regulator RcsA of *wca* gene expression, the phage shock protein encoding operon, and several genes (*spy*, *cpxP, slt, ycfS*) of cell envelope and nutritional (*cspD*, *csiD* and *yjiY*) stress responses. Since the RcsA-RcsB heterodimer activates the expression of the *wca* gene cluster and downregulates the expression of flagellar genes [15, 18], the downregulation of *rcsA* (one of the highest downregulated genes) could account for the downregulation of the *wca* gene cluster and upregulation of flagellar genes but not the other differentially expressed genes because *rcsB* was not differential expressed in the CR^+^ isolate.

In addition to the differential expression of genes implicated in biofilm formation and induction of stress responses, several genes encoding for transport systems (ABC-types transporters for putrescine, glutamine, leucine/isoleucine/valine, dipeptides, phospholipids and phosphate; permeases for N-acetylglucosamine; outer membrane porins OmpA and PhoE; transporters for glycerol-3-phosphate and γ-aminobutyrate; and cation: proton antiporters), biosynthesis of amino acids (isoleucine/valine, threonine, leucine, asparagine and histidine biosynthesis), amino acid degradation (γ-aminobutyrate), metabolism of intermediates of glycolysis (acetyl-CoA), TCA cycle (succinate), utilization of galactose, biosynthesis of cofactors (NAD, menaquinones, ubiquinones, vitamin B_12_) and biosynthesis of cell wall were downregulated in the CR^+^ isolate. This downregulation of a majority of differentially expressed genes was also apparent in the genes encoding hypothetical functions. On the contrary, a disproportionately small number of genes that were involved in the transport (nitrite/nitrate, D-serine, biotin, mannose, sugar phosphate), biosynthetic (lysine, aspartate/fumarate) and metabolic (sulfur, glucose and lipids) functions showed upregulation in the transcriptome of the CR^+^ isolate. These findings are different from those reported for a Congo red-binding variant of an *E. coli* O157:H7 meat isolate from a 1993 outbreak that showed increased biofilm formation and reduced resistance to different stresses but enhanced expression of genes for catabolic, metabolic and nutrient uptake pathways [17]. This suggests that the global transcriptional response resulting from the inactivation of the *rcsB* gene might differ between strains depending on the presence or absence of additional genes unique to these strains.

As described above, multiple two-component signal transduction pathways embedded in the bacterial cell membranes sense a variety of intracellular and extracellular signals to activate specific response regulators that alter the expression of specific sets of genes to ensure survival and adaptation of bacterial cells to the new environment. However, none of the genes constituting these signal transduction pathways were differentially expressed in the transcriptome of the CR^+^ isolate. Thus, either a differentially expressed but yet an uncharacterized gene (gene with a hypothetical function) or an inactivation of a known gene through a recombination or a mutagenic event might be responsible for the altered transcriptome of the CR^+^ phenotype. Comparative genomics of the CR^+^ and CR^−^ isolates revealed the presence of a tandemly duplicated 5-bp sequence that disrupted the ORF encoding the response regulator RcsB of the RcsF/RcsC/RcsD/RcsA-RcsB phosphorelay system [18]. The CR^+^ isolate complemented with the *rcsB* gene or the *rcsDB* operon from the CR^−^ parental strain displayed Congo red binding, biofilm formation, motility and acid resistance phenotypes (few of the phenotypes we selected for testing) similar to that of the CR^−^ parental strain. The complementation results thus indicated that the RcsF/RcsC/RcsD/RcsA–RcsB phosphorelay system is a major signal transduction system for controlling the switch in isolates unable to bind Congo red and produce biofilms to Congo red–binding and biofilm–producing isolates. In addition, many genes in the transcriptome of the CR^+^ isolate were downregulated without the downregulation of their known transcriptional regulators, suggesting that RcsB or RcsB-RcsA heterodimer might be involved in the regulation of the expression of these genes. Importance of RcsB in this switch is also evident in reports showing the presence of RcsB-binding sequences or boxes in the vicinity of promoters controlling the expression of genes required for biosynthesis of flagella, curli fimbriae and various enzymes and transporters linked to the acid stress response [15, 18, 84, 89]. A few studies have reported that inactivation of the *rcsB* gene by the deletion of either of the two insertion-sequence (IS) elements present in this gene or insertion of new IS elements within the preexisting IS elements, or deletion of the entire *rcsB* gene plus or minus adjacent sequences alters the biofilm-producing ability of the mutant isolates [17, 90]. The 5-bp duplication that we identified was present in the second IS element spanning nucleotides 403 – 458 of the *rcsB* gene in the CR^+^ isolate (NADC 6565). Thus, based on these studies, IS elements in the *rcsB* gene appear to serve as preferred sites for the genetic rearrangements leading to the inactivation of *rcsB* that is implicated in the regulation of pathways linked to bacterial survival and virulence.

## Conclusions

In this report, we showed that the disruption of the *rcsB* gene in a CR^+^ isolate resulted in the downregulation of majority of the differentially expressed genes implicated in transport, metabolism of carbohydrates, energy and amino acids. Overall, the CR^+^ isolates that arise from the CR^−^ strain, as a result of the *rcsB* inactivation, may provide a significant survival advantage for *E. coli* O157:H7, particularly in the environment. More specifically, the CR^+^ isolate we described in this report would be better suited for growth in nutrient-poor, pH neutral and low temperature environments, which are conducive for biofilm formation and reduced metabolism by not synthesizing a number of gene products that are not essential for growth and survival in natural environments. Slightly reduced growth rates that we observed at both 28 °C and 37 °C in rich and minimal media presumably correlates with the downregulation of many metabolic pathways in the CR^+^ isolate. In addition, increased sensitivity to acidic and oxidative stresses at 37 °C compared to at 28 °C might also be suggestive of better survival of CR^+^ isolate at lower growth temperatures via the formation of biofilms. Also, increased expression of *narK* (nitrate/nitrite antiporter) and *adhE* (aldehyde-alcohol dehydrogenase) in the CR^+^ isolate would promote fermentative metabolism for slow growth. Moreover, the *cspD* gene, a starvation-induced protein upregulated in the CR^+^ isolate, which is inhibitory to DNA replication, promotes formation of persister cells [91]. In conclusion, *rcsB* inactivation that gives rise to CR^+^ isolates may confer a unique set of adaptive advantages to *E. coli* O157:H7 isolates. Understanding the molecular basis of these adaptive events is important for development of strategies to mitigate carriage of these foodborne pathogens in carrier animals and reduce their survival in the environment.

## Abbreviations

CFU: Colony-forming units
CR: Congo red
DMEM: Dulbecco’s modified Eagles’ minimal medium
*Escherichia coli* O157:H7: O157
LB: Luria-Bertani
OD600: Optical density at 600 nm
TCST: Two-component signal transduction
YESCA: Yeast extract casamino acid
